# Risk and protective factors for relapse among Individuals with Schizophrenia: A Qualitative Study in Dar es Salaam, Tanzania

**DOI:** 10.1186/s12888-014-0240-9

**Published:** 2014-08-30

**Authors:** Adellah E Sariah, Anne H Outwater, Khadija IY Malima

**Affiliations:** Hubert Kairuki Memorial University (HKMU), Faculty of Nursing, 322 Regent Estate, P.O. Box 65300, Dar es Salaam, Tanzania; School of Nursing, Muhimbili University of Health and Allied Sciences (MUHAS), P.O. Box 65001, Dar es Salaam, Tanzania; Tanzania Commission for Science and Technology (COSTECH), P.O. Box 4302, Dar es Salaam, Tanzania

**Keywords:** Relapse, Schizophrenia, Caregivers, Risk factors, Protective factors, Tanzania, Africa

## Abstract

**Background:**

Relapse in people with schizophrenia is a major challenge for mental health service providers in Tanzania and other countries. Approximately 10% of people with schizophrenia are re-admitted due to relapse at Muhimbili National Hospital (MNH) Psychiatric Unit each month. Relapse brings about negative effects and it results in a huge burden to patients, their families, the mental health sector and the country’s economy. So far no study has been done to address relapse in Tanzania. The purpose of the study was to explore perspectives on risk and protective factors influencing relapse of people with schizophrenia and their caregivers attending Muhimbili National Hospital Psychiatric Out-patient Department, Dar es Salaam, Tanzania.

**Methods:**

A qualitative study was conducted, involving in-depth interviews of seven people with schizophrenia who are out-patients and their seven family caregivers at MNH. Purposive sampling procedure was used to select participants for the study. Audio recorded in-depth interviews in Swahili language were conducted with all study participants. The recorded information was transcribed and analyzed using NVivo 9 computer assisted qualitative data analysis software.

**Results:**

Personal risk and protective factors for relapse, environmental risk and protective factors for relapse and suggestions to reduce relapse were the main themes that emerged from this study. People with schizophrenia and their caregivers (all of whom were relatives) perceived non adherence to antipsychotic medication as a leading risk factor of relapse; other risks included poor family support, stressful life events and substance use. Family support, adherence to antipsychotic medication, employment and religion were viewed as protective factors. Participants suggested strengthening mental health psycho-education sessions and community home visits conducted by mental health nurses to help reduce relapse. Other suggestions included strengthening the nurse-patient therapeutic relationship in provision of mental health care.

**Conclusions:**

This study calls for improvement in mental health care service delivery to individuals with schizophrenia. Establishing a curricular in mental health nursing that aims to produce competent mental health nurse force would improve nursing practice in mental health care service delivery.

## Background

Schizophrenia is a disabling group of brain disorders characterized by symptoms such as hallucinations, delusions, disorganized communication, poor planning, reduced motivation, and blunted affect [1]. Schizophrenia is often accompanied by relapse even while on treatment [2]. Relapse has been defined as a worsening of psychopathological symptoms or rehospitalization in the year after hospital discharge [3]. Schizophrenia follows a variable course, with complete symptomatic and social recovery in about one-third of cases. Schizophrenia can however follow a chronic or recurrent course, with residual symptoms and incomplete social recovery. Individuals with chronic schizophrenia constituted a large proportion of all residents of mental institutions in the past and still do where these institutions exist [4].

Relapse may result in hospitalization, treatment resistance, cognitive impairment due to progressive structural brain damage personal distress incarceration and interference with rehabilitation efforts [5]. A few studies regarding relapse and schizophrenia have been done in Africa. Research in South Africa has found that presence of a co-morbid depressed mood, poor medication adherence due to a lack of patient insight and side-effects appear to be the factors most likely to increase the risk of a relapse [6].

Tanzania’s national hospital’s Department of Psychiatry, admits about 150 patients with different psychiatric disorders per month: 15 (10%) of these are readmissions due to relapse in schizophrenia. Apart from patients attending the clinic for follow-up, approximately 15 patients with specific psychiatric problems are attended per day within the department. The number of patients needing mental health and psychiatric care overloads the available mental health care team.

Most drugs available in the Psychiatric Unit are typical antipsychotics which have a lot of extrapyramidal side effects. Availability of atypical antipsychotics (most commonly Risperidone and Olanzepine) with lesser extrapyramidal side effects varies; hence patients have to buy the drugs for themselves. Sometimes they cannot afford to buy them due to poor social economic status which is made worse by the mental illness hence they go without medications which results into relapse. The structure of in-patient unit does not allow for a good and quality therapeutic nurse- patient relationship and communication. This kind of structure prevents nurse- patient interaction and hinders nurses’ exploration of patients’ problems and concerns which if identified in advance can lead to interventions that can perhaps help to reduce relapse.

Community mental health services are not well established. The absence of these services deprives the community from benefits of case management, outreach clinics, family visits, family therapies; counseling and other psychotherapies, which would also help reduce relapse in the community. Relapse in schizophrenia has a lot of effects to patients, care givers, the health sector and the country economy at large. Patients tend to deteriorate in their level of functioning with each relapse; hence their contribution to economic activities diminishes. Caregivers have to take care of the patient’s bills in the hospital once readmitted which becomes very costly. The health sector is imposed with a large burden and has to deal with the higher number of patients’ re-hospitalization. Therefore exploration of risk and protective factors for relapse among people with schizophrenia is a prerequisite in promoting mental health and preventing relapse in schizophrenia. However studies for schizophrenia especially on risk and protective factors for relapse are rare in Africa, including Tanzania. This study is intended to start addressing that need.

## Methods

### Design

A descriptive qualitative method was used to explore and aid understanding of the risk and protective factors for relapse in people with schizophrenia. Out-patients with schizophrenia and their caregivers were involved in the study.

### Setting

The catchment area of the study was in Dar es Salaam Region, Tanzania. The whole country has a population of about 43 million [7] and it is found in East Africa. Dar es Salaam region has a population of about 3.1 million people [8].

### Procedure

Sampling of participants to be enrolled in the study took place at MNH Psychiatric Outpatient Department (OPD). Sampling was done to the point at which no new information was obtained from participants and redundancy was achieved, i.e. data saturation [9]. Seven adult out-patients and their seven caregivers were purposefully selected for the study. Participants of diverse demographic characteristics (age, sex, marital status) were sought in order to obtain maximum variation. All patients had a diagnosis of schizophrenia according to DSM-IV criteria for more than 6 months and had a history of two or more psychiatric hospitalizations. With the help of the mental health nurse in-charge, participants were selected by going through files of patients with appointments that day, to identify those who met the study inclusion criteria.

Caregivers were the key guardian for the patient. They were 18 years of age and above; and had lived with the patient in the same household for more than 6 months. Selection of caregivers was done by identifying those who had escorted the selected patients to the OPD.

Those who agreed to participate were asked to provide their contacts such as phone numbers for subsequent booking of the appropriate day and venue for the interview. Participants who were ready to be interviewed on the same day, agreed to meet the researcher after having been attended by a psychiatrist. Patients who had come alone were asked to provide phone numbers for contacting their caregivers so as to book for the appropriate day and venue for the interview.

### Interviews

Eligible participants were given full explanation of the study, and its importance. They were also informed of the data collection procedures which involved audio recording of in-depth interviews (IDIs). All interviews were conducted in Swahili.

### For patient interviews, the topic guide was as follows:

- *What would you say are the reasons that you experience relapses*?
- *How does each of these reasons affect you*?
- *What assists you to cope with this illness that could protect you from relapse*?
- *How does each of these factors assist you to cope*?
- *What makes it difficult for you to cope with this illness*?
- *How does each factor make it difficult for you to cope*?
- *What suggestions would you like to make to the mental health nurses regarding relapse reduction or prevention*?

The topic guide for the caregivers was as follows:

- *What would you say are the reasons for your relative to experience relapses*?
- *How does each of these reasons affect him*/*her*?
- *What assists your relative to cope with this illness that could protect him*/*her from relapse*?
- *How does each of these factors assist him*/*her to cope*?
- *What makes it difficult for the patient to cope with this illness that could contribute to relapse*?
- *How does each factor make it difficult for him*/*her to cope*?
- *What suggestions would you like to make to the mental health nurses regarding relapse reduction or prevention*?

The interviews covered items in the topic guide and any additional material that was spontaneously suggested by participants. To gain maximum information, all participants were encouraged to give their own detailed personal account of relapse experience. Individual perceptions from participants, was obtained by interviewing patients and their caregivers separately, one after the other. Field notes were taken by the researcher throughout the interviews. The interviews ranged from approximately 30 to 45 minutes.

### Ethical considerations

Ethical approval was obtained from the ethical board of Muhimbili University of Health and Allied Sciences (MUHAS). Permission to conduct the study was obtained from the Executive Director of Muhimbili National Hospital (MNH), and subsequently the Head of Department, Psychiatry. Participants who agreed to participate in the study were requested to complete the written informed consent form, and arrangements for an interview with each potential participant were then made. Confidentiality and freedom to withdraw at anytime during the study was ensured.

### Data analysis

Demographic information was analyzed descriptively. Information from the field notes was incorporated into the data. The interview transcripts were examined in totality, to obtain an overall sense of the content of the responses by the participants to various issues. Audio-recorded interviews were transcribed to text computer files. The accuracy of the transcribed data was checked by listening to the recorded interviews. Minor mistakes were corrected.

Swahili transcripts were used for analysis to maintain the originality of the data. Content analysis of the data was done using NVivo 9 computer assisted qualitative data analysis software. Transcribed interviews (sources) were imported into the software for analysis.

### Trustworthiness

In describing trustworthiness the concepts of credibility, dependability and transferability were used [10]. Credibility in this study was covered in various ways:

1. Interviews were conducted until data saturation was achieved, this provided deeper information about the phenomenon under study;
2. Data triangulation [11] was used and it was achieved by investigating the patients’ files; and by interviewing people with schizophrenia and their caregivers about the same topic.
3. To ensure dependability [10], an interview guide was used to ensure consistency during data collection.

## Results

**Figure 1:**
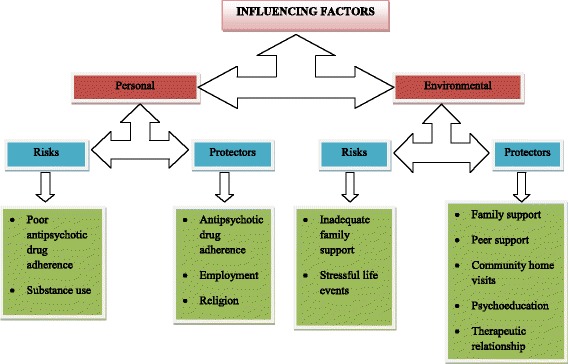
**Main nodes (categories) and subcategories of the influencing factors of relapse identified in this study.**

Participants’ characteristics can be seen on Table 1. Four major themes (personal and environmental risks and protectors) emerged from the data (See Figure 1).

**Table 1:** **Characteristics of patients and their caregivers at MNH**, **Psychiatric Clinic**

**Participants**	**Age**	**Sex**	**Education level**	**Occupation**	**Mental status**	**Relationship with @ other**	**Medication**	**Reported no of relapses**
P1	36	M	Secondary	College student	Single	Son	Modicate 25 mg 1/12	>5
C1	69	F	Primary	Retired nurse	Widow	Mother		>5
P2	40	M	Secondary	Nursery school teacher	Divorsed	Brother	Chlopromazine 100 mg nocte	>5
C2	37	M	College	Primary school teacher	Married	Brother		>5
P3	55	F	Primary	Peasant	Married	Sister inlaw	Haloperidol 1.5 mg OD	3
C3	45	F	Secondary	Bussiness woman	Married	Sister inlaw		3
P4	30	F	Primary	None	Divorced	Daughter	Haloperidol 1.5 mg Am & 3 mg nocte, Artane 5 mg OD	>5
C4	79	M	Primary	Retired	Widower	Father		>5
P5	56	F	Primary	Bussiness woman	Married	Wife	Haloperidol 3 mg BID	>5
C5	67	M	Primary	Mechanic	Married	Husband		>5
P6	25	M	Primary	Cook	Single	Son	Olanzepine 10 mg OD	3
C6	45	F	Primary	Food vender	Divorced	Mother		
P7	27	M	Secondary	Business man	Single	Brother	Haloperidol 1.5 OD	2
C7	31	F	Secondary	Business woman	Married	Sister		

Note: “P” stands for patient and “C” stands for caregivers.

### Personal risk factors for relapse

These are factors within the individual that increases the likelihood of experiencing relapse.

### Poor antipsychotic drug adherence

One of the main factors described by patients to have caused a number of relapses was poor adherence to antipsychotic medications. Non-adherence was due to the severity of drug side effects, forgetfulness, belief of cure, expense and lack of supervision.

All patients stated that the reason they sometimes do not take their medication is because of the drug side effects. Patients expressed their concerns about how drug side effects trouble them and disturb their normal lives.

A male patient, 40 years of age, experiencing muscular rigidity acknowledged:

….*I have problems with my legs*, *nowadays they become numb*, *they tighten. Even walking is difficult*……”

Caregivers also expressed their concern about the side effects of the antipsychotics. A male aged 37 years said about his brother:

*Many times he has been saying that*, "*I have a numbness problem*"

It was also said by several patients and some of the caregivers that at times patients forget to take their medications which in turn results into relapse. A lack of supervision and reminders were also found to be a cause for relapse. A 36 year old male patient said:

“…. *You find that I have forgotten to take my medication*, *somebody has to insist that I take medication*….”

Patients indicated that they stopped taking their medication after seeing that they are cured. This was supported by the fact that they no longer experienced the disease symptoms they had before. This belief led to stopping the medication and relapse followed. A 55 year old female patient, claimed:

“*Yes*, *I used medication*, *but when I felt that I was cured I stopped*…. *I was coming to the clinic but when I felt cured I stopped attending. I felt I was cured*.”

A patient’s mother, aged 45 years, expressed her concern about how atypical antipsychotics are expensive. Hence due to poor social economic status and lack of support from other relatives at times the patient is compelled to go without medications because the mother alone cannot afford to buy the medication. This leads to relapse.

*Yes they changed to other drugs which are too expensive*; *now we have to buy. When the drugs were finished*, *he went on without his medicine for a couple of months. The condition recurred because the financial situation was difficult*.

A 30 year old female patient also claimed that at times she stops taking medication because there is nobody to supervise her, despite the fact that she has been told about the importance of medication, frequency, and dosage.

*My father was at work at that time. There was nobody to supervise me*.

### Substance use

Smoking "cigarettes" (bhangi, local name for cannabis) was identified to be a precipitator of relapse by a 25 year old male patient.

*This problem started because I used to smoke* “*sigara kubwa*” (*the big cigarette*, *a local name for cannabis*).*Whenever I smoked*, *I became confused*.

### Anger

Patients and caregivers expressed their concern about how anger has resulted into relapse. A 56 year old female patient said:

“*When somebody gives you an unpleasant reply you become angry*…. *for example when somebody answers you rudely*…*I get angry. I carry it in my head and I get confused* (*nachanganyikiwa*).”

Another caregiver 31 years of age, reflected:

“*Whenever he got angry or whenever he was furious*, *that condition used to recur*.”

### Personal protective factors for relapse

Protective factors of relapse are individual factors that make it less likely for relapse to occur. The presence of such factors in an individual with schizophrenia was believed to reduce the risk of relapse in such individuals.

### Antipsychotic drug adherence

Apart from side effects, patients and their caregivers described the importance of adhering to antipsychotic medication and the role it plays in preventing relapse. A patient’s husband reflected:

“*She uses medication well. When she does so her condition improves*… *if she follows medication instructions*, *she gets no problems*.”

Patients and their caregivers also said that the use of long acting injectables, especially Modicate (Fluphenazine decanoate), helps to alleviate psychotic symptoms and reduce the number of relapses. These types of drugs also make it easier for them to adhere to the treatment regimen than the oral antipsychotics.

*Frankly at times I get confused*, *but after I have been injected*, *all the things I had done before come to an end. I truthfully go to school*, *I learn and come back home. I participate in several activities at home*.

The patient’s mother supported this by saying:

“*When he uses Modicate*, *we seldom come to the hospital due to disease symptoms*.”

### Religion

Patients stated that their religious activities help to improve their condition. A 36 years old male patient said:

“*When I go for the choir I interact with my fellow choir singers*; *it usually helps me to be active*, *and you find yourself okay just like other people*.”

Another male patient aged 40 years added:

“*For sure it is because I am a very religious person*; *therefore*, *I take my religion book and read. It strengthens perseverance*.”

### Employment

Caregivers claimed that when patients engage themselves in different economic activities to earn a living they feel free to spend their income because they do not have to ask others for financial support. This boosts their self esteem makes them independent and prevent psychological problems which may precipitate relapse. It also gives them the ability to support their families. A patient’s husband expressed:

“*Ever since her youth she is not somebody who would sit and wait to be served*, *she is a person who works*….. *When she does her work she feels free*.”

This was supported by another patient’s sister in-law caregiver who said:

“*She is a peasant*…*being a peasant helps her because firstly*, *it keeps her busy. She does not have the time to sit and think*.”

A 25 year old male patient expressed that his job helps him to earn a small amount of money to cater for their basic needs, such as medication which helps reduce disease symptoms and prevent relapse episodes.

*It helps in those days that I do not get medicine from the hospital*; *I get to buy my own medicine*.

### Environmental risk factors for relapse

Several environmental factors were also viewed to have negative effects on patients’ recovery. Such factors in the environment trigger for the return of psychotic symptoms.

### Inadequate family support

Some patients complained about criticism from their caregivers and that they are not supported in times of need. This tends to disturb them psychologically and as a result they tend to relapse due to the lack of support in treatment regimen. A 40 year old male patient claimed:

“………*I followed him five times*, *I told him*, “*Uncle*, *I am sick. I am very sick and I cannot get in a bus. It is very far from Mbagala to Muhimbili. I cannot go on my own*, *I need somebody to hold my hand because standing in a bus is very difficult for me. So I ask you to help me and take me to the hospital*.” *But he refused*, *just like that*.”

One female caregiver (patient’s mother) expressed her concern about how it is difficult for her and the patient to get support from other relatives. This lack of support especially financially, makes it difficult for the caregiver and patient to obtain medication whenever they are required to purchase. As a result the patient relapses because of failure to obtain medication. She complained:

*“It is difficult to make a decent life*……*his father is already dead and his relatives on his father*'*s side* (*upande wa kiumeni*) *nobody does anything to help or support him*.” (*She cried silently while wiping off her tears*).

### Stressful life events

One patient declared that she had experienced a number of relapses manifested by recurrence of symptoms after having a miscarriage and also after losing a baby during birth.

*The first time I was pregnant and I got a miscarriage at three months*, *I started to get confused* (*nilivurugikiwa*)……. *After the miscarriage then I found as if I was confused mentally and I saw as if the earth was changing*…….*I became fine but then I got pregnant again*, *I was brought to Muhimbili*, *I gave birth but the baby did not survive*, *I was confused once again*.

One female patient, aged 56 years, claimed that one of the reasons she experiences relapse is when she thinks about things she has not accomplished in life.

*At times I think*; *I have not even built my own hut* (*kibanda*). *I then think about what I should do so that I can get my hut*, *I think*, *I want my children to be like this or I want my husband to be like this*…

The patient’s husband added:

“*Her problem was about thinking about things which she is not certain to get*….*and when she does not get them*; *it disturbs and confuses her* (*anachanganyikiwa*)…..”

### Environmental protective factors for relapse

Environmental protective factors are within the individuals’ surroundings that tend to make it less likely that relapse will occur. Participants gave different views on factors in the environment that tended to protect them from relapse; these included family and peer support, and mental health services.

### Family support

More than half of all patients indicated that the presence of support and love from their family, neighbors and the community reduced the number of relapse episodes. A 36 year old male patient declared:

“*On my family*'*s side for example*, *honestly they all care for me very much. They don*'*t like to see me reach a state of confusion. That is why they bring me to the hospital when they see that I have reached a bad state*”

This was supported by a female patient aged 55 years old.

“*Ah*! *My relatives help me so much. Firstly those relatives are the ones who saved me*, *because I did not know myself*……*I had been suffering*….*They show a lot of love. They do stigmatize me*. (*kunyanyapaa*) *a patient*.”

### Peer support

Peer support was shown to play a big part in keeping the patient better and also in protecting the patient from relapse. One patient aged 25 years declared:

“*I have friends*, *who comfort me and support me. Whenever I get sick*, *many of them come to visit*.”

A female caregiver (patient’s sister) said:

“*I noticed that his friends help him because they have not discriminated against him*…..*When he is with them they talk like they used to prior to the illness*.”

### Mental health services

The majority of patients and their care givers confirmed that the mental health services they receive help a lot. They acknowledged that, If those services were unavailable the condition of the patient would not have improved.

“*Mental health services help a lot. Good services begin from when we receive mental health education up to when they provide us with medication*.”

### Suggestions to reduce relapse

Several suggestions were made by participants to reduce relapse. The main suggestion was the improvement of mental health services with much emphasis on community home visits, strengthening of provider-patient therapeutic relationship, and psychoeducation.

### Community home visits

Patients suggested that substantial recovery will occur and relapses could be prevented if mental health nurses followed them to their homes in the community to know the progress of their illness. Community services would yield clear information about the condition of the patient. The course of the illness would be monitored and the cause of their illnesses would be identified. One patient said:

“*For example I think I would suggest that mental health nurses should make follow up at our homes because waiting here brings ambiguity* (*utata*) *because the patient says this and the caregivers say that. Therefore it is difficult for one to know what to follow*”

The patient’s mother added:

“*It would be good if you* (*mental health nurses*) *would visit us at our homes. That would be very good. You would talk to the patients*, *find out how he has been since the other day*, *and how one feels*.”

A 37 year old male caregiver (patient’s brother) suggested that mental health nurses should educate families with mentally ill patients on the risk factors to mental illness.

“*When it is known that this problem has been inherited*, *then those in the family should be called. So they can be told that this problem is in the clan and it can happen to anyone*.”

Caregivers urged that mental health workers should try their best to get enough time and talk to the patients and caregivers. They also suggested that at times it would be beneficial to communicate with them through cell phones; that way they could urge them to come or to set appointments for such talks for the betterment of the patient’s condition.

“……..*What I would like to be done is for the patients and their caregivers to get enough time for mental health counseling*.”

### Provider-patient therapeutic relationship

A 45 year old female caregiver (patient’s sister in-law) suggested that the time the patients spends with a psychiatrist when they come for follow up is very short. It does not allow the mental health provider to talk and listen to the patient's concerns and needs. The mental health nurses, physicians or psychiatrists should spend enough time with patients to get to know their mental conditions.

“….*If you talk with the patient like this you get to know the patient rather than when the patient arrives*; *you just take the card*, *you write eeeh how have you been*!, *you write tatatata*.....*come at a certain date*…. *But when you take time to talk to the patient*, *I think you can know more about his or her condition*.”

A patient’s sister recommended that all mental health workers should try their best to use pleasant language when interacting with clients when they come to seek mental health care because the language itself is therapeutic to the patient.

“*I am asking you to educate each other that when you take care of somebody a pleasant language is preferred even before you provide medication to that person*.”

## Discussion

The study highlighted relapse concerns facing schizophrenic patients and their caregivers in Dar es Salaam. The findings suggested that both patients and caregivers perceived non adherence to antipsychotic medication as a leading factor of relapse. Others included inadequate family support, stressful life events and substance use. Adherence to antipsychotic medication, family and peer support, employment and religion were perceived to protect patients from relapse episodes. Psychoeducation, community home visits and a good therapeutic relationship could help reduce relapse and promote mental health in patients with schizophrenia.

### Risk factors for relapse

#### Non adherence to antipsychotic medication

Non compliance to antipsychotics was demonstrated to be an important factor that resulted in relapse of several patients. Previous studies have also found that non adherence appears to be one of the factors most likely to increase the risk of relapse in schizophrenic patients [6]. Medication noncompliance and under-compliance continues to be a problem in the treatment of schizophrenia in the United States and Canada; the vast majority of hospital admissions for exacerbation of psychosis have been linked to noncompliance [12]. In Germany it has been found that patients who tend to experience a relapse are less likely to have a positive attitude toward treatment adherence [3].

The majority of patients in this study were on typical antipsychotic medication and they reported at least one side effect that was at least somewhat bothersome. Typical antipsychotic drugs are likely to produce exrapyramidal side effects and akathisia, which may directly lead to non-adherence [13]. Patients in Dar es Salaam reported sedation and exrapyramidal side effects such as rigidity, and tremors, which is consistent with prior research [14,15]. Taking medication daily is a burden for these individuals, which is made worse by the side effects of these drugs. In an effort to get rid of these discomforts, individuals stop taking their regular medication which in turn causes them to experience relapse. Providing education with emphasis on the importance of medication would help these individuals to be responsible of the medication management process.

The interviews of half of patients revealed lack of ongoing assessment. These patients had stopped attending the psychiatric unit for follow up visits, several times. They had also stopped taking medication believing their symptoms had resolved [16] and that they were cured.

Lack of awareness among families about the nature of the illness also contributes to discontinuation of treatment. In India when the symptoms were controlled and the patient had recovered sufficiently to become functional, he was seen as being "cured" and no longer in need of treatment [17].

#### Inadequate family support

Low perceived social support among caregivers has been associated with relapse among mentally ill clients [18]. The family factor is one of the psychosocial factors that affects the clinical course and outcome of schizophrenia including its recurrence. Social support deficits are risk factors for non-adherence in schizophrenia. Persons who live alone may lack medication supervision and have difficulty accessing medical care. Lack of support from family might hinder persons with schizophrenia in achieving rehabilitation [13]. Poor family support in individuals with mental illness may be evidenced by clients being denied food, laughed at and even beaten at other times [19].

Family members may not support the client because they would not want to share some of the property the patient owns. Hence they tend to ignore the patient’s symptoms and treatment a practice which results into relapse [20].

#### Stressful life events

Several patients from the study reported that unfavorable events in their lives have in one way or another contributed to a number of relapses in their lives. These events included loss of a child during birth, miscarriage, being angered by care givers, and criticism from care givers. These results are consistent with previous studies that have found that patients with schizophrenia are more sensitive and more susceptible to the negative effects of even minor stressors. Stressful life events are often associated with the onset of a psychotic relapse, usually in the 3 weeks prior the relapse [6].

One patient expressed his concern on how he received critical comments from his care givers and how these comments made him feel. It has been shown that patients from families with high criticism had a significantly higher number of psychiatric readmissions and longer cumulative length of stay at psychiatric hospitals than patients from families with low criticism [21]. High criticism has been found to be associated significantly with poorer outcome and a worse course of illness than those who did not feel criticized [21]. The results are also consistent with a study that was done by [22]. Patients with schizophrenia expressed increased stress relating to their domestic environment, which was a result of interpersonal conflicts between patients and their parents, children, neighbors or extended family members.

#### Substance use

Cannabis use was found to be a factor that was believed by one patient to have precipitated relapse. Studies in Germany have also shown that second to alcohol, cannabis is the most frequently misused substance among patients with schizophrenia [23]. In patients with an established psychotic disorder cannabis abuse is associated with a higher risk of psychotic relapses and with poorer social outcome. Cannabis is also frequently abused by schizophrenic patients, and it is associated with worse clinical out-comes [24]. Given that psychoactive compounds within cannabis can cause or increase psychotic experiences secondary to intoxication effects [25] it is very plausible that cannabis might lead to increased positive symptoms and subsequently relapse or re-hospitalization in people with psychosis.

It has also been suggested that schizophrenic patients are more vulnerable to the effects of tetrahydrocannabinol, which is the main psychotropic compound of cannabis. Cannabis use has been reported to increase delusions and hallucinations in people with schizophrenia, while the findings regarding negative symptoms have been contradictory [26].

### Protective factors for relapse

#### Family and peer support

Importance has been given to the family environment as a contributing factor to the relapse or rehabilitation of the patient. The family is an important factor which affects the patient’s mental well-being and outcome. Support from family promotes adherence in patients with mental illness which aids recovery. The kind of support they provide to clients include medication, supervision, monitoring the drug intake and taking the patients to mental health facilities regularly [27].

The family support networks were reported as strong by more than half of the participants in this study. Patients were very grateful for the support they received from their care givers during their illness episodes, when attending the clinic for follow up visits, encouragement and supervision when taking their medication and in fulfillment of other basic needs of life like education, employment, and health in general. These results are in agreement with a study of 121 patients and their 121 family members in Japan which found that more than 70% of the families replied that they ‘often’ or ‘sometimes’ supported the patients in mental health management and daily living such as ‘observation of the condition’ and ‘taking drugs’ [28].

Helping families to maintain and enhance a supportive social network may represent a useful means to reduce family burden in schizophrenia [29]. People with mental illness receive critical support from family members. Support and reciprocity with family members are important elements thet relates to the patient’s recovery process [30]. Caregivers appreciated the support they received from other relatives. Family support tends to reduce the burden that caregivers may have to experience in caring for patients with schizophrenia [31].

#### Drug adherence

Antipsychotic medication compliance was expressed by patients and their care givers to be a strong protector of relapse. It was found that the relapse risk was substantially lower when a patient was adhering properly to the antipsychotic therapy [12]. Improvement in symptoms and the recognition of this improvement by patients may have led to improvement in their attitude to medication [12]. Furthermore, patients’ subjective attitude toward medication is associated with treatment adherence and objective measures of symptom responsiveness among individuals with schizophrenia.

Some patients reported fewer antipsychotic drug side effects than others. Those who were on atypical antipsychotic drugs such as Olanzepine are among patients who were adherent to medications. This is consistent with a study which found that fewer side effects, as well as the effectiveness of atypical antipsychotic drugs in managing psychotic symptoms, make it more likely that persons will continue their treatment. Thus, the choice of antipsychotic drug affects adherence [13].

#### Religion

Religion was indicated by three patients and their caregivers to have beneficial impacts in the course of their illness. They added that religion, including singing in church choir, creates a sense of belonging, enables them to deal with difficult situations and gives them the strength to move on despite their mental conditions. There is a growing amount of literature suggesting that religion and spirituality may provide positive coping to patients with schizophrenia [32]. Promotion of mental health through religion has been found to be as important as treatment of mental illness. Religion fosters recovery even when symptoms persist and people with mental illnesses can be helped to achieve fulfilling, meaningful and purposeful lives [33].

Religion and spirituality can play an important part in recovery. Religious activities have been shown to be helpful in dealing with psychiatric symptoms. Individuals with mental illness who experience greater symptom severity and lower overall functioning engage more in religious activities as part of coping with mental problems [34] which enhances recovery.

The positive impact of spirituality on adherence to treatment is explained by an improved quality of life, a better social support, and more positive representations of the illness by believers. Religion affects the self and may improve recovery by instilling hope, purpose, and meaning in life but also affects the adherence to treatment [35].

#### Employment

This study also found that when patients have a job to earn income they become independent. This boosts their self esteem and helps them feel that they can contribute something to the family or society. The importance of having meaningful employment has been emphasized in reflecting patients’ needs to reach personal goals, hopes, and aspirations, as important components of well-being. This is believed to contribute to both increased self-esteem and to better management and reduction of symptoms including negative, positive, and depressive symptoms [36]. Having a job tends to keep patients busy; they tend not to think of their mental illness. Employment has repeatedly been shown to have an important role in patients’ recovery from any severe mental illness, particularly schizophrenia [37].

Employment not only provides income, it also improves activity and social contacts for patients with schizophrenia. It fosters financial benefits; provide coping strategies for psychiatric symptoms, and ultimately facilitating the process of recovery from mental illness [37].

### Suggestions to reduce relapse

#### Therapeutic relationship

Therapeutic factors within therapeutic relationships relate to how the patient reacts to the interventions offered by the nurse. This reaction is typified by the notion of rapport between the patient and the nurse. The rapport refers to the affinity and emotional closeness between two individuals and how this is recognized and used in a therapeutic way [38].

Relationships have been shown to play an important role in the development of trust [39]. When clients receive good services they develop trust, which fosters a sense of belonging and a good relationship, leading to positive motivation regarding care [40]. Other studies have found that a good therapeutic relationship between patients with schizophrenia and mental health professionals is important for adherence to antipsychotic medication [41].

Individuals with mental illness often experience periods of despair related to psychiatric symptoms or psychosocial consequences of having a psychiatric illness. The presence of someone who can provide hope and empathy towards an individual’s suffering helps through these difficult times [42].

Patients and their caregivers from this study suggested that mental health nurses and psychiatrists should dedicate their time to listen to their concerns so as to improve patients’ conditions. However due to shortage of well trained mental health nurses at the psychiatric unit, this kind of relationship may be poorly formulated.

#### Community home visits

Patients and their caregivers emphasized the importance of home visits by mental health nurses. They urged that regular visits would enable them to receive psychoeducation on patient management, drug adherence and side effects. Regular psycho-education programs must be conducted in the community to educate the families about the nature of their relative's illness and the need for sustained medical treatment [17]. Community visits have been shown to help patients deal with their symptoms, promote relaxation, and help clients take medication regularly, improve client’s self-care abilities and improve interactions between clients and families [43].

#### Psychoeducation

The purpose of patient education/teaching (or psychoeducation) is to increase patients’ knowledge and understanding of their illness and treatment. It is supposed that increased knowledge enables patients with schizophrenia to cope more effectively with their illness [44]. Patients were concerned that medications only would not reduce the problem of relapse. They urged mental health nurses to also provide more counseling and psychoeducation sessions which would help improve their condition. Patients who understand their illness, medications, and treatment expectations consistently demonstrate better adherence [45].

Caregivers suggested the importance of educating families with mentally ill individuals on risk factors of mental illness so as to protect those who have not developed symptoms. Psychoeducation programs for patients, families, and caregivers aimed at coping with schizophrenia have been shown to improve adherence, reduce substance abuse, reduce relapse, and shorten hospital stays [46].

These findings call for improvement in service delivery for mentally ill individuals especially health education sessions on the importance of medication adherence and follow up. Further emphasis should be focused on training of more mental health nurses who will provide quality mental health services for inpatient care, outpatient care, and primary mental health care which meet the needs of a larger number of individuals with mental illnesses in Tanzania.

#### Limitations of the study

The results should be transferred with caution as recruiting caregivers who had accompanied patients to the Psychiatric Out-patient Department probably contributed to inclusion of caregivers with very good relationship with their patients and interest in patient care. They might have also given socially desired answers. However the atmosphere during the interview indicated honesty. In addition, the small sample may limit the transferability of the findings particularly in relation to ethnicity and social economic conditions. A larger sample of participants with diverse characteristics could have resulted in different perspectives of relapse experience.

## Conclusions

This study highlighted the perceptions of patients with schizophrenia and their caregivers about risk and protective factors of relapse. The findings call for improvement in mental health care service delivery to individuals with schizophrenia. Establishing a curricular in mental health nursing that aims to produce competent mental health nurse force would improve nursing practice in mental health care services. These results can be exploited in influencing health policy makers to improve mental health and reduce the burden of relapse in individuals with schizophrenia, their families and community as a whole. In addition, the results can help establish clear policies articulating measures to reduce relapse in patients with schizophrenia and then develop systematic plans with dedicated budget and agreed timelines.

## Abbreviations

DALYs: Disability adjusted life years
HKMU: Hubert Kairuki Memorial University
IDIs: In-depth Interviews
MNH: Muhimbili National Hospital
MOHSW: Ministry of Health and Social Welfare
MUHAS: Muhimbili University of Health and Allied Sciences
NBS: National Bureau of Statistics
OPD: Out Patient Department
WHO: World Health Organization
